# Inhibition of apoptosis signal-regulating kinase 1 enhances endochondral bone formation by increasing chondrocyte survival

**DOI:** 10.1038/cddis.2014.480

**Published:** 2014-11-13

**Authors:** G J Eaton, Q-S Zhang, C Diallo, A Matsuzawa, H Ichijo, M J Steinbeck, T A Freeman

**Affiliations:** 1 Department of Orthopaedic Surgery, Thomas Jefferson University, Philadelphia, PA 19107, USA; 2 Department of Spine Surgery, The Second Xiangya Hospital of Central South University, Changsha, Hunan 410011, PR China; 3 Laboratory of Cell Signaling, University of Tokyo, Tokyo 113-0033, Japan; 4 Open Innovation Center for Drug Discovery, University of Tokyo, Tokyo 113-0033, Japan; 5 School of Biomedical Engineering, Science & Health Systems, Drexel University, Philadelphia, PA 19104, USA

## Abstract

Endochondral ossification is the result of chondrocyte differentiation, hypertrophy, death and replacement by bone. The careful timing and progression of this process is important for normal skeletal bone growth and development, as well as fracture repair. Apoptosis Signal-Regulating Kinase 1 (ASK1) is a mitogen-activated protein kinase (MAPK), which is activated by reactive oxygen species and other cellular stress events. Activation of ASK1 initiates a signaling cascade known to regulate diverse cellular events including cytokine and growth factor signaling, cell cycle regulation, cellular differentiation, hypertrophy, survival and apoptosis. ASK1 is highly expressed in hypertrophic chondrocytes, but the role of ASK1 in skeletal tissues has not been investigated. Herein, we report that ASK1 knockout (KO) mice display alterations in normal growth plate morphology, which include a shorter proliferative zone and a lengthened hypertrophic zone. These changes in growth plate dynamics result in accelerated long bone mineralization and an increased formation of trabecular bone, which can be attributed to an increased resistance of terminally differentiated chondrocytes to undergo cell death. Interestingly, under normal cell culture conditions, mouse embryonic fibroblasts (MEFs) derived from ASK1 KO mice show no differences in either MAPK signaling or osteogenic or chondrogenic differentiation when compared with wild-type (WT) MEFs. However, when cultured with stress activators, H_2_O_2_ or staurosporine, the KO cells show enhanced survival, an associated decrease in the activation of proteins involved in death signaling pathways and a reduction in markers of terminal differentiation. Furthermore, in both WT mice treated with the ASK1 inhibitor, NQDI-1, and ASK1 KO mice endochondral bone formation was increased in an ectopic ossification model. These findings highlight a previously unrealized role for ASK1 in regulating endochondral bone formation. Inhibition of ASK1 has clinical potential to treat fractures or to slow osteoarthritic progression by enhancing chondrocyte survival and slowing hypertrophy.

Apoptosis signal-regulating kinase 1 (ASK1) is a protein at the apex of the mitogen-activated protein kinase (MAPK) stress response pathway that determines cell fate, survival and death decisions. ASK1 is activated by reactive oxygen species (ROS), reactive nitrogen species (RNS), endoplasmic reticulum release of calcium (ER stress), receptor activation by cytokines or disrupted integrin binding.^1, 2, 3, 4, 5^ As such, it is responsible for directly translating intracellular ROS into downstream effects, and excessive activation or dysregulation of ASK1 signaling can have pathogenic results. Most importantly, as a key determiner of cell death, inhibition of ASK1 increases overall cell survival, thereby decreasing degenerative processes after injury. As ASK1 activation is also important for inflammatory cell migration and activation, inhibition leads to an amelioration of the inflammatory response, a potential contributor to adverse outcomes in some disease processes.^6^ Thus, the inhibition of ASK1 has been reported to decrease the progression and severity of several diseases, including cardiovascular and neurodegenerative diseases.^7, 8^

ASK1 is constitutively produced by all cells and exists in the cytoplasm as an inactive complex bound to inhibitory proteins thioredoxin, glutaredoxin or 14-3-3.^9, 10, 11, 12^ In the presence of oxidative stress, disulfide bond formation occurs, accompanied by a conformational change of the inhibitory proteins and their dissociation from ASK1.^9^ This dissociation allows the self-dimerization, autophosphorylation and activation of ASK1.^4, 13^ Activated ASK1 selectively phosphorylates c-Jun N-terminal kinase (JNK) and/or p38 MAPK to initiate signaling cascades that control the aforementioned cytokine and growth factor signaling, cell cycle regulation, cellular differentiation, survival, hypertrophy and apoptosis.^14, 15, 16^

Oxidative stress is known to have a role in bone formation, growth and remodeling of adult bone.^17, 18, 19, 20^ Specifically, oxidative stress drives the process of hypertrophy and death of terminally differentiated chondrocytes in the growth plate, which is necessary for long bones to lengthen and form properly. Chondrocyte death is thought to be initiated through caspase-3 (CASP3), and a report by Hatai, *et al.* ^21^ implicates ASK1 as an upstream activator of mitochondria-dependent CASP3 activation. In addition, JNK and p38 MAP kinase pathways are activated by ASK1 and both have important roles in chondrogenesis, bone formation and turnover. Specifically, activated JNK enhances chondrogenesis by activating the transcription factor activator protein-1 (AP-1), which upregulates the expression of transforming growth factor beta (TGF*β*).^22^ Whereas, prolonged p38 activation is required for the maturation and differentiation of chondrocytes and it is necessary for endochondral ossification.^23^ For bone, JNK acts as a repressor of osteogenesis,^24^ but both p38 and JNK are required for receptor activator of nuclear factor kappa B ligand (RANKL)-induced osteoclastogenesis and bone resorption.^25^

While the ASK1 knockout (KO) mouse was generated in 2001,^26^ no skeletal phenotype has been reported, despite the high expression of ASK1 in wild-type embryonic mouse chondrocytes.^26, 27^ This is most likely due to the lack of obvious gross phenotypical changes. In this report, we have assessed the role of ASK1 in skeletal tissues during development and in skeletally mature mice using microCT and histology. To do this, we employed the ASK1 KO mouse and a small molecule inhibitor of ASK1 in both *in vivo* and *in vitro* models of bone development and endochondral ossification. We found that inhibition of ASK1 activation accelerated diaphyseal bone formation, increased the amount of trabecular bone and enhanced bone volume in an ectopic endochondral ossification model. These results highlight the potential of ASK1 inhibition as a potential therapeutic option to enhance fracture healing and prevent osteoarthritic progression by enhancing chondrocyte survival and slowing hypertrophy.

## Results

### Accelerated diaphyseal bone formation and increased trabecular bone volume in ASK1 KO and heterozygous mice

**Figure 1:**
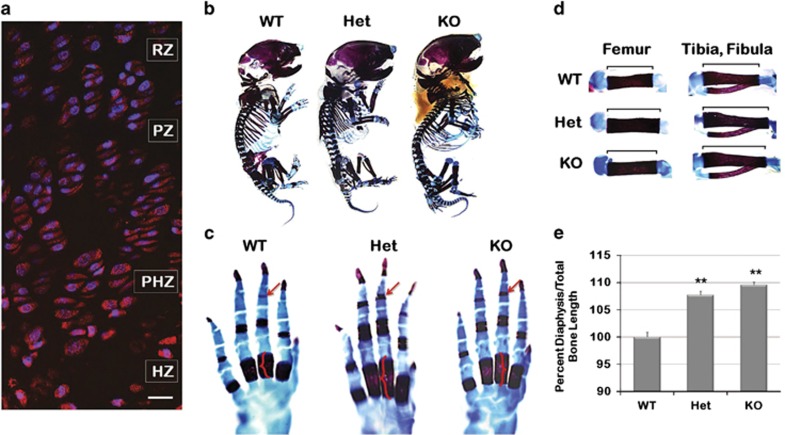
Accelerated bone formation in developing KO mice. To determine whether the loss of ASK1 would affect embryonic bone growth, the skeletal phenotype was evaluated using whole mount staining, histology and micro-computed tomography (microCT) of WT, Heterozygous (Het) and KO mice littermates. (**a**) ASK1 localization in the growth plate of PD14 mice showed increased expression in the hypertrophic zone (resting zone, RZ; proliferative zone, PZ; prehypertrophic zone, PHZ; hypertrophic zone, HZ). (**b**) PD1 WT, Het and KO mice stained with alcian blue and alizarin red revealed the KO was slightly larger. (**c**) Earlier calcification in the distal phalange (arrows) and greater mineralization (brackets) of the diaphysis of the metacarpal were present in the Het and KO mice. (**d**) Measurement of alcian blue and alizarin red stained femur, tibia and fibula (not shown: humerus, radius, ulna) from WT, Het and KO mice confirmed a significant increase in Het and KO diaphyseal length as compared with WT (**e**). (*n*=4 animals for each genotype; *P≤0.05; **P≤0.01; scale bar, 50 *μ*m)

No gross skeletal phenotype for the ASK1 KO mouse has been reported; however, a close examination of the skeletal phenotype had not been performed before this study. To determine whether the loss of ASK1 would affect embryonic bone growth, the skeletal phenotype was evaluated using whole-mount staining, histology and micro-computed tomography (microCT) of WT, heterozygous (Het) and KO mice littermates. Immunostaining for ASK1 also demonstrated it was highly expressed throughout the growth plate of WT mice, with an increased cytoplasmic concentration in the hypertrophic zone (Figure 1a). Whole-mount staining of each post-natal day 1 (PD1) mouse skeleton with alizarin red (mineral deposition) and alcian blue (cartilage formation) revealed the KO was slightly larger (Figure 1b; *n*=4 per genotype). Closer evaluation of the metacarpals and phalanges showed increased length of the diaphyseal bone of the metacarpals (Figure 1c, brackets) and mineralization of the distal most phalange in Het and KO mice (Figure 1c, arrows). To further investigate accelerated bone development, the diaphyseal length per total bone length (diaphysis and epiphysis) was determined for the femur, tibia, fibula, humerus, radius and ulna and compared as percent of WT. The results confirmed a significantly increased diaphyseal length in the Het (7.7%, *P*<0.0001) and KO (9.6%, *P*<0.0001), as compared with WT (Figures 1d and e).

**Figure 2:**
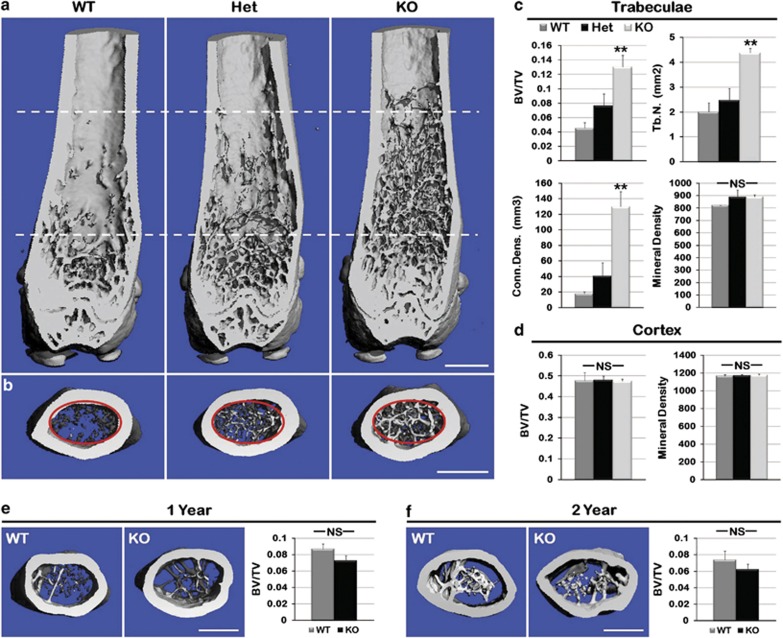
Increased trabecular bone in KO mice. To ascertain whether differences in bone formation persisted as the mice aged, 3-month-, 1–year- and 2-year-old mouse femurs from WT, Het and KO were subjected to microCT analysis. (**a**) Representative microCT generated longitudinal sections of hind limb femurs demonstrated increased number and penetration into the diaphysis of trabeculae (dotted lines) in the KO animal at 3 months. (**b**) Increased trabeculae observed in 200 16-*μ*m slices in cross-section of the KO mouse. (**c**) Analysis of trabecular bone (red circle in **b**) shows a significant increase in mineralized bone volume/total bone volume (BV/TV), trabecular number (Tb.*n*.) and connectivity density (Conn.Dens., an assessment of the number of connected structures in the trabecular bone network) in KO mice compared with WT. There was no difference in bone quality (mineral density) of trabeculae. (**d**) Cortical bone BV/TV and mean density showed no significant difference. (**e**) Trabecular analysis by microCT at 1 year of age showed equalization of BV/TV, which persisted through 2 years (**f**). (*n*=3 for each genotype; **P≤0.01; NS, not significant; scale bar, 500 *μ*m)

To determine whether enhanced bone formation persisted, 3-month-, 1-year- and 2-year-old mouse femurs from all three genotypes were analyzed using microCT. MicroCT longitudinal evaluation revealed Het and KO mice displayed visibly increased penetration of the trabeculae into the central diaphysis of the femur at 3 months (Figure 2a; *n*=3 per genotype). Cross-sectional analysis of 200 slices of the femur (Figure 2b, red circles) indicated a significant increase in trabecular bone volume/total volume (BV/TV) (Figures 2c, *P*=0.004), number of trabeculae (*P*=0.002) and connectivity density (*P*=0.002) in the femurs of Het and KO when compared with WT femurs (Figure 2c). No significant differences were observed in the cortical bone BV/TV or the quality of bone (bone mineral density; BMD) between the three genotypes (Figure 2d). Additionally, no differences were observed in trabecular or cortical bone of femurs from 1- and 2-year-old KO and WT mice (Figures 2e and f), indicating intramembranous bone formation is not affected by the lack of ASK1.

### Longer hypertrophic and shorter proliferative zones are present in the growth plates of Het and KO mice

**Figure 3:**
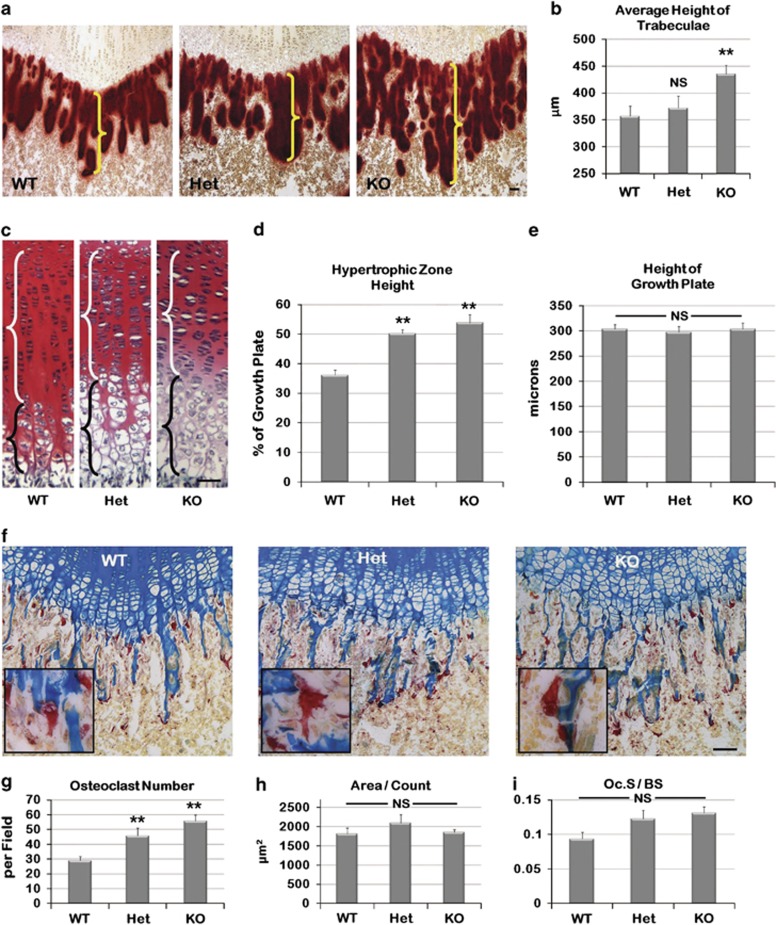
ASK1 KO histological examination of bone and cartilage. (**a**) Alizarin red staining of femurs from WT, Het and KO at PD14. (**b**) Analysis confirmed a significant increase in trabecular length in the KO mouse. (**c**) Saffranin O staining of the growth plates showed an increase in the length of the hypertrophic zone (black brackets), but a decrease in the proliferative zone (white brackets) for the Het and KO mice. (**d**) The significant increase in the length of the hypertrophic zone of both Het and KO mice expressed as % of growth plate. (**e**) The overall length of the growth plates is similar. (**f**) TRAP staining of growth plate from WT, Het and KO at day 14. (**g**) Analysis showed that the number of osteoclasts present was increased in Het and KO mice. (**h**) The size of osteoclasts was not significantly increased. (**i**) The osteoclast surface to bone surface ratio (Oc.S/BS) was not significantly increased. (*n*=10 limbs each genotype; *P<0.05; **P≤0.01; NS, not significant; scale bar, 50 *μ*m)

To determine the cellular dynamics responsible for the increase in diaphyseal length, WT, Het and KO mouse femurs from PD14 were sectioned and stained with alizarin red (mineralization), alcian blue (proteoglycan of cartilage) and tartrate-resistant acid phosphatase (TRAP, osteoclast marker; *n*=10 per genotype). Results from the alizarin red staining showed that Het and KO mice trabeculae were longer than those in the WT mice (Figures 3a and b, *P*=0.005), but there was no apparent difference in the total mineral deposition. Comparison of the femoral growth plates showed an increase in the length of the hypertrophic zone in Het and KO animals (Figure 3c, black brackets, D, *P*<0.0001, *P*<0.0001, respectively), but a decrease in the proliferative zone (Figure 3c, white brackets). The opposite differences in the length of these two zones ultimately resulted in no overall change in growth plate length (Figure 3e). Finally, osteoclast parameters, including osteoclast number, area and osteoclast surface on bone surface (Oc.S/BS), were determined on the equivalent femur sections for all three genotypes (Figure 3f). Both the Het and KO mice presented statistically significant increases in the number of osteoclasts (Figure 3g, *P*=0.007, *P*<0.0001, respectively), while there was no significant difference in either the osteoclast area or the Oc.S/BS measurements (Figures 3h and i).

### Chondrogenic and osteogenic potential are unaffected by the loss of ASK1 in culture

**Figure 4:**
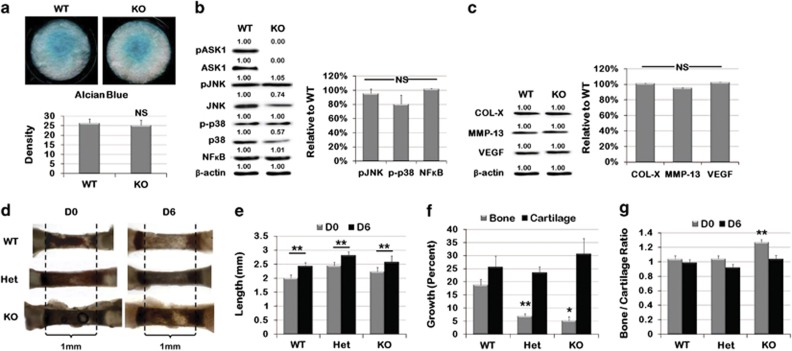
*In vitro* culture of Het and KO MEFs and metatarsals showed no difference in differentiation or growth as compared with WT. (**a**) Alcian blue staining of MEFs generated from WT and ASK KO mice, seeded as micromasses and cultured in chondrogenic media for 13 days showed no difference in density. (**b**) Western blots for chondrogenic WT and ASK1 KO MEFs showed no alteration in ASK1 KO downstream activation of JNK, p38 or NF*κ*B. (**c**) No difference was observed in COL-X, MMP-13 or VEGF expression, which are markers of chondrogenic differentiation. (**d**) Metatarsals are shown from PD5 WT, Het and KO mice after being isolated and grown in culture for 6 days. (**e**) Het and KO metatarsals were longer than WT at both D0 and D6, but all metatarsals grew in culture. (**f**) Analysis of compartmentalized growth showed that Het and KO metatarsal bone had less growth than WT, but cartilage growth was unaffected. (**g**) Analysis of bone to cartilage ratio showing ASK1 KO metatarsals had a high bone to cartilage ratio at D0, but assumed the WT ratio of 1.0 by D6. (NS, not significant; *P<0.05; **P<0.01; micromass: *n*=12 wells for each genotype; Western: *n*=4 replicates; metatarsals: *n*=12 for each genotype)

Further investigation into how the loss of ASK1 affects chondrogenic differentiation was determined by culturing MEF cells derived from WT or KO mice in chondrogenic media for 13 days. No significant differences were found in alcian blue staining of MEFs after 13 days (Figure 4a; *n*=12 per genotype). To determine whether the lack of ASK1 affected the activation of JNK and p38, the total and phosphorylated protein amounts were determined by western blot analysis. No differences were observed in either total or activated JNK or p38 (Figure 4b; *n*=4 per genotype). In addition, NF*κ*B, a well-known transcription factor regulating chondrocyte differentiation, also showed no differences between genotypes.

To determine the expression of proteins typically associated with hypertrophic chondrocyte differentiation, we assessed expression of collagen X (COL-X), matrix metalloprotease-13 (MMP-13) and vascular endothelial growth factor (VEGF). No significant difference between WT and KO was observed for any of these markers (Figure 4c). Similar results were observed in WT and KO MEFs cultured in osteogenic differentiation media based on the expression levels of osteoblast markers: alkaline phosphatase (Alk Phos), bone morphogenic protein 4 (BMP4), bone sialoprotein (BSP) and runt-related transcription factor (RUNX2) (Supplementary Figure 1).

### Restoration of cartilage to bone ratio in Het and KO metatarsals in culture

To further explore the effect of ASK1 on cartilage, growth and bone deposition, WT, Het and KO metatarsals from PD5 mice were isolated and placed in organ culture for 6 days (Figure 4d; *n*=12 per genotype). Metatarsals were imaged daily and the length of both the whole-metatarsal and the diaphysis was measured. After 6 days in culture, all metatarsal bones grew and total metatarsal length at D6 was not significantly different (Figure 4e). However, calculation of the percent growth of the diaphysis showed significant differences; the WT diaphyseal bone grew 18.76%, Het grew only 6.98% and KO 5.19% (Figures 4f, *P*<0.0001, *P*=0.0105, respectively). To investigate this discrepancy, the ratio of bone to cartilage was calculated at both D0 and D6 (Figure 4g). At D0 the KO had significantly greater bone to cartilage with a ratio of 1.2, compared with 1.0 in the WT (*P*=0.0015), but by D6 the bone to cartilage ratio of all genotypes had stabilized at 1.0. Taken together, the MEF differentiation and metatarsal growth results suggest that under tissue culture conditions the loss of ASK1 does not significantly affect skeletal cell or tissue behavior.

### Stress-induced retardation of bone growth is not observed in Het and KO metatarsals

**Figure 5:**
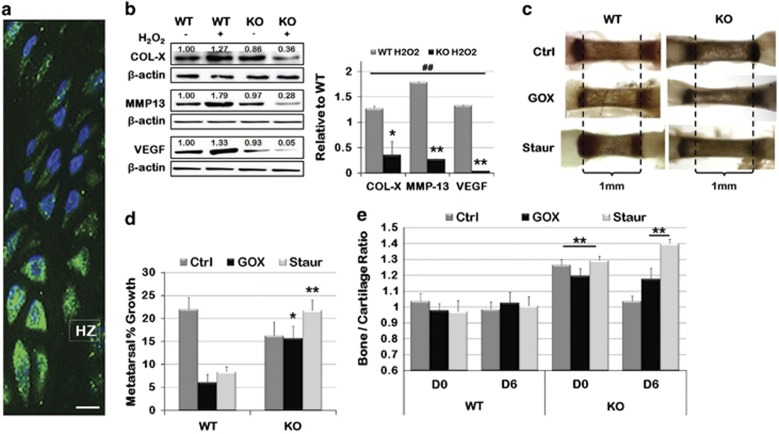
Addition of ASK1 activators to *in vitro* culture slowed differentiation of Het and KO MEFs and stabilized metatarsal growth compared with WT growth impairment. (**a**) Nitrotyrosine fluorescent immunohistochemistry showed intense staining in the hypertrophic zone of the PD14 growth plate. (**b**) WT MEF micromasses cultured in chondrogenic media for 13 days with H_2_O_2_ showed an increase in chondrogenic differentiation markers by Western blot, while KO MEFs showed a decrease. (**c**) Representative PD5 WT and KO metatarsals incubated with GOX (to generate H_2_O_2_) or Staur in the culture medium for 6 days. (**d**) WT metatarsals incubated in GOX or Staur showed a reduction in growth compared with control, but KO metatarsals showed no reduction in growth. (**e**) The bone to cartilage ratio of KO metatarsals was maintained after 6 days in the presence of GOX or Staur treatment, while in control media KO metatarsals reverted to the ratio observed in WT metatarsals. (*n*=12 for each genotype; *P<0.05; **P<0.01; ^##^*P*<0.01 to WT Ctrl; scale bar, 50 *μ*m)

Normal development and terminal differentiation of chondrocytes takes place in the presence of oxidative stress, which is known to activate ASK1. Figure 5a shows hypertrophic chondrocytes stained with an antibody for nitrotyrosine, a marker for proteins modified by the presence of ROS and reactive nitrogen species (RNS). As these conditions are not present in the tissue culture environment, we added H_2_O_2_ or staurosporine (Staur) to the KO and WT MEF culture media. In the presence of these stressors, markers of hypertrophic differentiation (COL-X, MMP-13 and VEGF) were all enhanced in WT MEFs, but significantly decreased in the KO (Figure 5b).

Similarly, WT metatarsals cultured in the presence of glucose oxidase (GOX), which generates a constant amount of H_2_O_2_, or Staur showed a decreased elongation of the diaphysis compared with untreated WT metatarsals (Figure 5c). When percent growth of the metatarsals was calculated, WT growth showed significant inhibition in the presence of GOX (6.0%) or Staur (8.4%), while growth of the KO metatarsal displayed equal (15.7% in GOX) or increased (21.7% in Staur) growth as compared with the KO-untreated control (Figures 5d, *P*=0.045, *P*=0.002, respectively).

Investigation of the bone to cartilage ratio, comparing D0 to D6, showed the WT metatarsals consistently maintained a 1.0 ratio, regardless of treatment. While the KO control initially displayed a bone to cartilage ratio of 1.2 (GOX) or 1.4 (Staur) at D0, and a 1.0 by D6 (Figures 5g, *P*=0.01, *P*<0.0001). These data suggest that the presence of stress is required to obtain the phenotype observed *in vivo* and highlights the role of ASK1 activation in the growth plate.

### Decreased death pathway signaling is observed in KO MEFs as compared with WT

**Figure 6:**
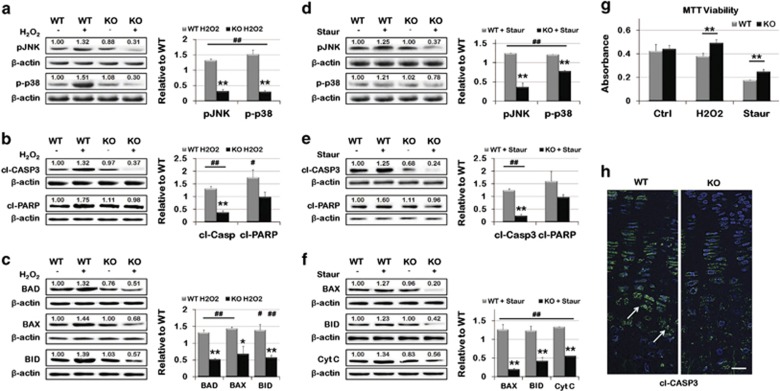
Altered death signaling in KO MEFs in response to stress. (**a**) Twenty-four hours culture with H_2_O_2_ caused increased phosphorylation of JNK and p38 in WT MEFs, both were decreased in ASK1 KO cells. (**b**) An increase in cl-CASP3 and cl-PARP, both apoptotic activators, was also observed in WT MEFs, but not in KO MEFs. (**c**) Less expression of the apoptotic effectors BAD, BAX and BID was observed in KO cells, in contrast to their upregulation in WT cells. (**d**) For KO cells cultured in the presence of Staur, decreased phosphorylation of JNK and p38 was observed. (**e**) Decreased cl-CASP3 and cl-PARP were also observed in KO MEF Staur culture. (**f**) Similarly, BAX, BID and cytochrome c release were reduced in KO MEFs cultured with Staur. In each case, the opposite was seen in WT MEFs. (*n*=3 replicates; *P<0.05; **P<0.01; ^#^*P*<0.05 to WT Ctrl; ^##^*P*<0.01 to WT Ctrl) (**g**) An MTT viability assay showed that WT cells have significantly decreased viability compared with ASK1 KO cells after 24 h culture with H_2_O_2_ or Staur (*n*=4 replicates; **P<0.01). **(h)** Immunohistochemistry revealed decreased cl-CASP3 in the growth plate of KO mice, compared with WT mice. (Scale bar=50 *μ*m)

During the process of hypertrophy, chondrocytes in the growth plate are exposed to stress followed by apoptotic cell death. To investigate whether loss of ASK1 protects chondrocytes from death, MEFs isolated from WT and KO mice were seeded as a micromass and cultured in chondrogenic media containing H_2_O_2_ or Staur. WT chondrogenic MEFs treated with H_2_O_2_ displayed a significant increase in ASK1 signaling, as evidenced by increased phosphorylation of JNK and p38 (Figure 6a). Conversely, JNK and p38 phosphorylation after H_2_O_2_ stimulation were decreased in KO MEFs. A significant decrease in cleavage of both CASP3 and Poly (ADP-ribose) polymerase (PARP), two initiators of apoptosis, was also observed (Figure 6b). Furthermore, decreased expression of the apoptosis effectors Bcl-2-associated death promoter (BAD), Bcl-2-associated X protein (BAX) and BH3 interacting-domain death agonist (BID) was observed in the KO MEFs (Figure 6c). Similarly, treatment with Staur showed significant decreases in ASK1 signaling in the KO MEFs compared with WT (Figure 6d), concurrent with a decrease in apoptotic proteins cl-CASP3, cl-PARP (Figure 6e), BAX, BID and cytochrome c (Figure 6f).

Finally, an MTT viability assay showed a decrease in cell death in KO MEFs compared with WT when treated with either H_2_O_2_ or Staur (Figure 6g). This is consistent with a decrease in cl-CASP3 throughout the growth plate of PD14 KO mice and increased nuclear localization in hypertrophic chondrocytes of WT femurs (white arrows; Figure 6h).

### ASK1 inhibition increased bone volume in an ectopic ossification model

**Figure 7:**
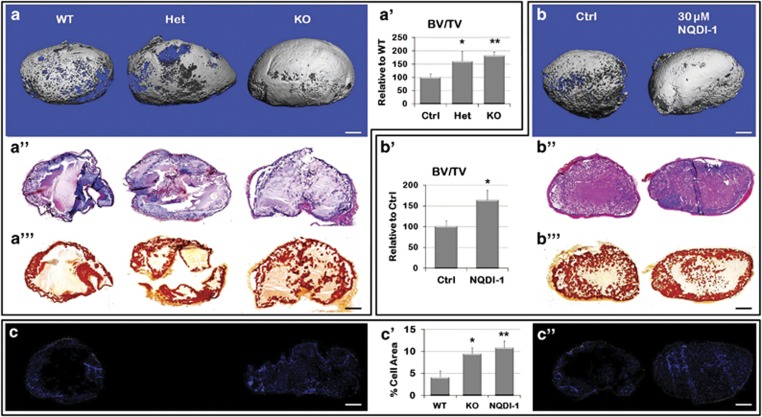
Ectopic ossification recapitulates endochondral ossification in KO or ASK1 inhibited WT mice. (**a**) Matrigel /BMP ectopic masses formed in WT, Het and KO mice 2 weeks post-implantation analyzed by microCT. (**a**′) Het and KO mice showed a 60% and a 82% increase in BV/TV, respectively, compared with WT mice. (**a**′′) Serial sections of the ectopic mass stained with alcian blue and H&E for cartilage visualization or (**a**′′′) alizarin red for mineral deposition. (**b**) Matrigel /BMP ectopic masses with 30 *μ*M of the ASK1 inhibitor NQDI-1, increased BV/TV as compared Matrigel /BMP alone (**b**′) Ectopic masses with NQDI-1 showed a 64% increase in BV/TV above WT. (**b**′′) Increased alcian blue and H&E staining of serial sections of the ectopic mass indicated more cartilage (**b**′′′) and alizarin red staining was increased indicating more bone deposition. ASK1 KO (**c**) and NQDI-1 treated (**c**′′) DAPI stained ectopic mass sections showed an increase in the number of cells as compared with WT. (**c**′) Quantitation of cell number increase in the KO and NQDI-1 ectopic mass as compared with WT. (*n*=15 WT, 9 Het, 19 KO, 14 NQDI-1; *P≤0.05; **P≤0.01; scale bar, 1 mm)

To remove the bias of the developmental process in endochondral bone formation in the growth plate, we used an ectopic model of endochondral ossification in 8-week-old mice of each genotype. Matrigel (serving as a support scaffold)/rhBMP-2 were injected subcutaneously into the ventral abdominal wall and ossification of the ectopic mass was measured after 2 weeks. An increase in the bone volume was observed with the loss of ASK1 based on three-dimensional microCT reconstructions of representative ectopic masses (Figure 7a). In accordance, an increased BV/TV ratio was also observed in Het (60.4% *P*=0.03) and KO mice (81.7% *P*=0.001), as compared with WT mice (Figure 7a'). There was also an increase in alcian blue (Figure 7a'') and alizarin red staining indicated more cartilage and newly formed bone (Figure 7a'''). No differences were observed in the bone mineral density of the ectopic masses based on genotype.

To determine whether a small-molecule inhibitor could recapitulate the results observed in the KO mouse, we used NQDI-1, a selective inhibitor of ASK1.^28^ The optimal inhibitor concentration range was determined using 10 *μ*M, 30 *μ*M and 50 *μ*M on isolated WT MEFs, (Supplementary Figure 2a). Ten micromolar NQDI-1 reduced ASK1 phosphorylation to 40% of control and reduced the phosphorylation of JNK and p38 (Supplementary Figure 2b). NQDI-1 at 30 *μ*M almost completely and 50 *μ*M completely prevented ASK1, JNK and p38 phosphorylation. We then tested the efficacy of both 25 *μ*M and 50 *μ*M on bone formation using the Matrigel/rhBMP ectopic ossification CD-1 mouse strain model. In this model, 25 *μ*M increased bone volume, whereas 50 *μ*M NQDI-1 decreased ossification, as compared with WT (Supplementary Figures 2c and d). Alcian blue and alizarin red staining confirmed that both chondrogenesis and osteogenesis were enhanced (Supplementary Figures 2e and f).

To confirm this result in C57BL/6 mice, we added 30 *μ*M NQDI-1 to the BMP/Matrigel mixture before injection into WT mice and repeated the ectopic ossification experiments. After 2 weeks, this concentration of NQDI-1 was sufficient to increase bone volume 64% above control (*P*=0.04) (Figures 7b and b'), which was comparable to the 82% increase observed in the KO. No differences were observed in the BMD of the treated and control ectopic masses. We also confirmed that both chondrogenesis and osteogenesis were enhanced, based on the increased alcian blue and alizarin red staining with respect to control (Figures 7b'' and b'''). Finally, we determined the number of cells in each ectopic bone sample using DAPI staining (Figures 7c and c''). Both the KO mouse, and the WT with NQDI-1 showed increased cell counts in the bone forming tissue (Figures 7c', *P*=0.02, *P*=0.01, respectively). This trend was also observed as early as 1 week post implantation (data not shown).

## Discussion

The purpose of this study was to investigate the involvement of ASK1 signaling in skeletal cell differentiation, development and death. Herein, we show that the absence (ASK1 KO mouse) or inhibition (ASK1 inhibitor, NQDI-1) of ASK1 enhances hypertrophic chondrocyte survival resulting in increased endochondral bone formation. Specifically, mice deficient in ASK1 displayed an increased hypertrophic zone of the growth plate, accelerated long bone mineralization and increased trabecular bone formation. The enhanced bone formation was not limited to embryonic long bone development, but was also observed during ectopic endochondral ossification in adult ASK1 KO mice and WT mice treated with NQDI-1. Interestingly, MEFs from the ASK1 KO mouse cultured *in vitro* showed no differences in MAPK signaling or osteogenic/chondrogenic differentiation under non-stress conditions. However, when stressed by H_2_O_2_ and staurosporine, the lack of ASK1 resulted in decreased activation of death signaling pathways and extended chondrocyte survival. Taken together, these results underscore a previously unrealized role for ASK1 in chondrocyte death and regulation of endochondral ossification, and highlight ASK1 inhibition as a possible therapeutic option to enhance fracture healing.

Previous characterization of ASK1 expression in WT adult and embryonic mice shows high expression in hypertrophic chondrocytes, but no obvious skeletal phenotype in the ASK1 KO mouse.^26, 27^ In fact, the ASK1 KO mice appear completely normal, are born at the expected Mendelian frequency and are indistinguishable in appearance from their WT littermates.^26^ Indeed, the accelerated diaphyseal bone formation and increased trabecular volume and length we observed required systematic analysis with microCT and histology. Closer examination revealed that hypertrophic chondrocytes in ASK1 KO mice displayed an increased resistance to death, which increased the length of the HZ (based on morphology). This in turn, resulted in accelerated long bone mineralization and increased trabecular bone deposition. While this appears chondrocyte specific, crosstalk between skeletal cells at the chondro-osseous junction in the ASK1 KO may influence this behavior.

This role for ASK1 in the growth plate is novel, but the loss of other proteins affecting hypertrophic chondrocyte apoptosis has also been reported to increase HZ length.^29, 30^ Specifically, mice lacking the death activating TNF*α* receptor 1 (TNFR1) show a similar phenotype. ^31^ As TNFR1 activates ASK1, this suggests the TNF pathway may be a primary driving force in hypertrophic chondrocyte death. Interestingly, when knocking out the death and apoptosis protein CASP3 in chondrocytes the opposite results are observed, and include delayed ossification, decreased bone mineral density and a shorter HZ,^32^ indicating the ASK1 death pathway may be as important, or more so, than CASP3. Osteoblastogenesis and osteoclastogenesis in CASP3 KO and ASK1 KO mice also show opposite behaviors as both are impaired in the CASP3 KO and increased in the ASK1 KO mice.^33^ However, as both mice were total KOs, there is no way to truly discern the individual contribution of each cell type to the observed phenotypes. Separate conditional KOs in chondrocytes, osteoblasts and osteoclasts would be required to determine their individual contributions.

Most other reports detailing the function of ASK1 have determined the presence of stress is required before a phenotype is uncovered. ^34, 35, 36^ Similarly, it was only in culture conditions where H_2_O_2_ or staurosporine was present that the ASK1 KO mouse MEFs or metatarsals showed decreased cell death and increased survival. Cells lacking ASK1 also showed a decrease in the basal levels of several death-associated proteins involved directly or indirectly with ASK1, which may indicate a feedback control that contributes to their resistance to apoptotic stimuli and cell death. These results emphasize the tightly controlled activation of ASK1 by the inhibitory binding of redox-sensitive molecules like thioredoxin, glutaredoxin or 14-3-3. Our results, together with the extensive literature on the role of ASK1 in mediating cell death in a multitude of cell types strengthens our conclusion that the lack of ASK1 slows the death of hypertrophic chondrocytes, lengthening the HZ allowing more deposition of trabecular bone during development.

To address the therapeutic potential of inhibiting ASK1 to promote bone formation, we used the ASK1 inhibitor NQDI-1 with a murine ectopic ossification model in two strains of mice and showed increased endochondral bone formation. This model is routinely used to characterize the basic biological properties of a given therapy, as straightforward analysis of the *in vivo* activity of cells is provided at specific stages of tissue repair.^37^ This model relies on the activation of an inflammatory response to the Matrigel injection to get mesenchymal cell invasion. Although not explored in this report, ASK1 activation haas a role in immune cell activation and production of pro-inflammatory cytokines;^36, 38^ however, ASK1 inhibition did not impede the required inflammatory response for mesenchymal cell invasion in our study. A possible explanation is that, while inflammation is essential for bone formation, excessive inflammation (excessive and/or prolonged production of pro-inflammatory cytokines) causes delayed bone formation.^39^ In support of this, our data suggest that the increased bone formation was due to an increase in the number of cells (either through increased migration into the Matrigel or enhanced proliferation), which ultimately increased chondrogenesis and ossification. Therefore, we can speculate that dampening the inflammatory response may have also contributed to the increase in endochondral bone formation.

Finally, if the ASK1 inhibitor is used therapeutically the question of bone quality in the presence of ASK1 inhibition is a concern. However, the results from the KO mouse indicate there is no difference in bone quality between the KO and the WT, so we would not expect bone quality (strength and stiffness) to be either improved or reduced. Taken together, this report highlights the potential of ASK1 inhibition as a therapeutic option to promote endochondral bone formation during fracture healing through increased bone formation, shortened repair time and improved healing of nonunions.

## Materials and Methods

### Cell culture

Cells were cultured in Dulbecco's Modified Eagle's Medium (DMEM (Thermo Fisher, Waltham, MA, USA)) containing 100 units/ml penicillin, 100 *μ*g/ml streptomycin (Cellgro, Manassas, VA, USA), 5% fetal bovine serum (FBS, (CellGro)), and 5% fetal calf serum (Gemini, West Sacramento, CA, USA) in a 5% CO_2_ incubator at 37 °C. The following treatment concentrations were used: H_2_O_2_ ((Thermo Fisher) 200 nM), Staur (Staur (Sigma, St. Louis, MO, USA), 1 nM), ASK1 inhibitor (NQDI-1 (Tocris, Bristol, UK), at 10 *μ*M, 30 *μ*M, and 50 *μ*M).

### Micromass culture

Culture of WT and KO MEF micromasses was performed as previously described.^40, 41^ In brief, cells were plated in 24-well plates at a density of 1 × 10^6^ cells per droplet in the center of each well. They were allowed to attach for 3 h, after which 150 *μ*l of DMEM was added to each well, supplemented with 2% FBS. For chondrogenic differentiation, ascorbic acid (Sigma, 50 *μ*g/ml), TGF*β* (Gemini, 10 ng/ml), Insulin-Transferrin-Selenium (ITS, (Gibco, Grand Island, NY, USA), 10 *μ*l/ml) and dexamethasone (Sigma, 10 nl/ml) were used in the differentiation media. For osteogenic differentiation, ascorbic acid (Sigma, 50 *μ*g/ml), *β*-glycerophosphate (BGP, Sigma, 10 *μ*l/ml) and dexamethasone (Sigma, 10 nl/ml) were used in the media. Media was changed every other day for 13 days.

### Western analysis

Cells were lysed in Mammalian Protein Extraction Reagent (MPER, Thermo Fisher), and protein concentrations were measured using Bio-Rad Protein Assay (Bio-Rad Laboratories, Hercules, CA, USA). Approximately 40 *μ*g of protein was loaded onto each lane of an sodium dodecyl sulfate (SDS)–polyacrylamide gel, and after electrophoresis the proteins were transferred to a polyvinylidene difluoride (PVDF) membrane. The membrane was blocked by incubation in Tris Buffered Saline (TBS) with 0.05% Tween 20 (Thermo Fisher, Waltham, MA) and 5% Membrane Blocking Agent (GE Healthcare, Buckinghamshire, UK) for 1 h while shaking. The membranes were then incubated with their respective primary in TBS with 0.05% Tween 20 overnight at 4 ^o^C. Antibodies used for western blot included: rabbit anti-pASK1, NF*κ*B, MMP13, VEGF and BAD, and mouse anti-*β*-actin and BAX (Santa Cruz Biotechnology, Dallas, TX, USA); rabbit anti-JNK, COL-X and BID (Abcam, Cambridge, MA, USA); rabbit anti-ASK1, pp38 and cl-PARP (Cell Signaling, Danvers, MA, USA); rabbit anti-pJNK, BSP and cl-CASP3 (Millipore, Billerica, MA, USA); rabbit anti-p38 and mouse anti-RUNX2 (Invitrogen, Carlsbad, CA, USA); mouse anti-BMP4 (R&D Systems, Minneapolis, MN, USA); and mouse anti-Cyt C (BD Bioscience, Franklin Lakes, NJ, USA). The primary antibody was removed and the blots were washed three times in TBS with 0.05% Tween 20. Then their respective horseradish peroxidase (HRP)-conjugated secondary antibodies (Santa Cruz) were applied to the blots which were incubated for 1 h at room temperature, washed intensively in TBS with 0.05% Tween 20 and then reacted with ECL Advanced Detection system (Amersham, Pittsburgh, PA, USA) for 5 min at 25 °C. Detection of the membranes was done with a FujiFilm Intelligent Darkbox (FujiFilm, Tokyo, Japan).

### Cell viability assay

The number of viable cells were evaluated by a colorimetric 3-(4,5-dimethylthiazol-2-yl)-2,5-diphenyltetrazolium bromide (MTT) assay. MEFs were seeded in 24-well plate at a density of 4 × 10^4^ cells/well, and cultured for 24 h to allow their adhesion to the plate. After pre-incubation, the culture medium was changed to experimental medium for 24 h: H_2_O_2_ (Thermo Fisher, 200 nM), Staur (Sigma, 1 nM). MTT reagent (Molecular Probes, Life Technologies, Grand Island, NY, USA) was added and incubated for an additional 4 h at 37 °C. The blue formazan crystals of viable cells were dissolved using isopropanol and the optical density (OD) was evaluated spectrophotometrically at 570 nm.

### Animals

ASK1 deficient (ASK1 KO) mice and C57BL/6N wildtype mice were used in the present study. ASK1 mice were backcrossed onto the C57BL/6N (Charles River Laboratories) background for at least 10 generations to reduce genetic variation. ASK1 KO mice were purchased from the Oriental Yeast Co., Tokyo, JP, with the permission of Dr Hidenori Ichijo. NIH guidelines for the care and use of laboratory animals were observed, and all animal protocols were approved by the Institutional Animal Care and Use Committee (IACUC) at Thomas Jefferson University.

### Whole-mount embryo staining and analysis

Whole post–natal day (PD) 1 littermates of WT, Het and KO ASK1 mice were killed after which the skin was removed; they were eviscerated and fixed in 95% ethanol for 4 days, followed by whole-mount staining with alcian blue (Thermo Fisher), for cartilage and alizarin red (Sigma Aldrich, St Louis, MO, USA) for bone, as previously reported.^42^ In brief the soft tissue was then dissolved in 1% potassium hydroxide (KOH) for 3 days allowing visualization of bone and cartilage. After staining the specimens were imaged and the diaphyseal length per total bone length (diaphysis and epiphysis) was determined for the femur, tibia, fibula, humerus, radius and ulna using ImagePro Plus (Media Cybernetics, Silver Spring, MD, USA). The results are reported based on the diaphyseal length per total bone length of the WT set to 100%.

Metatarsals complete with phalangeal joint were isolated from D5 mice as previously reported.^43^ They were then cultured for 6 days in DMEM containing 2% fetal bovine serum, 1% Penicillin/Streptomycin and 1% Amphotericin B (Gemini) at 37 °C. glucose oxidase (GOX (Sigma Aldrich) 10 mU/ml, to generate H_2_O_2_), or Staur (1nM) treatments were added to the media, which was changed daily. Images of the metatarsals were taken each day and total growth, bone growth and cartilage growth were measured using ImagePro Plus software (Media Cybernetics).

### MicroCT analysis of trabecular bone

Each knee joint was harvested, fixed in 4% paraformaldehyde (Sigma) and subjected to microCT analysis (Scanco *μ*CT 40; Basserdorf, Switzerland). The scans were performed in the long axis of the diaphysis, with an energy of 70 kVp, a current of 114 *μ*A and a 200-ms integration time producing a resolution of 12 *μ*m^3^ voxel size. Each scan comprised a minimum of 800 slices encompassing the knee joint, femur and tibia. For trabecular analysis, 200 slices of the femur above the patella were traced and contoured inside the cortex. The 3D data sets were low-pass filtered using a Gaussian filter (*σ*=1.0, support=2) and segmented with a fixed threshold filter (165 mg HA/cm^3^) according to the current guidelines.^44^ The following morphometric parameters of the trabeculae were calculated: trabecular bone volume fraction (BV/TV), that is, the ratio of trabecular bone volume over endocortical total volume, trabecular number, mineral density and connectivity density index, which calculates the number of trabecular connections per unit volume.

The knee joints were then decalcified, embedded in paraffin and 6 *μ*m sagittal histological sections were taken through the joint at 80 *μ*m intervals, stained with alizarin red (Sigma Aldrich) or Safranin O (Matheson Coleman & Bell, East Rutherford, NJ), and imaged on an Optiphot microscope (Nikon, Melville, NY, USA) with a Spot Color Camera (Diagnostic Imaging, Sterling, MI). To detect osteoclasts, staining for tartrate-resistant acid phosphatase (TRAP) was performed as described previously^33^ using a Leukocyte Acid Phosphatase Kit (Sigma Aldrich) and counter-stained in hematoxylin (Thermo Fisher). Histomorphometric analyses were performed on the stained slides to quantify the osteoclasts per bone perimeter.^17, 45^ Only multinucleated, TRAP-positive cells (red staining) on the bone surface were considered to be osteoclasts.

### Immunohistochemistry

Tissue slides were deparaffinized, rehydrated, then subjected to antigen unmasking solution (Vector; Burlingame, CA, USA) for 10 min at 100 °C. Slides were then washed with phosphate buffered saline (PBS). Next, all slides were incubated with 0.5% Triton X (Sigma) in PBS for 10 min at room temperature for permeabilization. After washing with PBS, the tissues were then blocked in 4% bovine serum albumin (BSA, Equitech-Bio, Kerrville, TX, USA) with 0.1% Tween 20 in PBS for 1 h at room temperature. Next, cleaved CASP3 antibody (Millipore), ASK1 antibody (Cell Signaling, Danvers, MA, USA), was diluted 1 : 50 or nitrotyrosine antibody (a generous gift from Harry Ischiropoulos, University of Pennsylvania, Philadelphia, PA) was diluted 1 : 200 with 1% BSA with 0.1% Tween 20 in PBS, placed on the slides and allowed to incubate overnight at 4 °C. Fluorescence immunohistochemistry was used to test for the prevalence of cleaved CASP3, ASK1, and nitrotyrosine in D14 mouse growth plates. A negative control sample was incubated without primary antibody and processed. Following primary antibody incubation, slides were washed in PBS and incubated for 1 h at room temperature with an appropriate Alxa-fluor 488-conjugated donkey anti-rabbit secondary antibody (Invitrogen). Following a final PBS wash, the slides were mounted with Vectashield Hard Set with 4',6-diamidino-2-phenylindole (DAPI, Vector) and imaged on an Eclipse E800 microscope (Nikon) with an Evolution QEi Monochrome camera (Media Cybernetics).

### Mouse ectopic endochondral ossification assay

An experimental model of endochondral ossification was utilized by induction of ectopic bone formation, as previously described.^46^ Specifically, mixtures of Matrigel (BD Bioscience) containing 3.5 *μ*g/ml of recombinant human bone morphogenetic protein-2 (rhBMP-2; Gene Script, Piscataway, NJ, USA) were prepared on ice, and aliquots (300 ml) were injected in up to four subcutaneous abdominal sites in 2-month-old CD-1 or C57BL/6 mice following IACUC-approved protocols. For inhibition of ASK1 in WT mice, 25, 30 or 50 *μ*M of the inhibitor NQDI-1 was added to the mixture. After 2 weeks, the resulting ectopic bone masses were dissected, fixed in 4% paraformaldehyde and subjected to microCT analysis. The scans were performed using an X-ray tube energy of 45kVp, a current of 177 *μ*A and a 200-ms integration time producing a spatial resolution of 12 *μ*m^3^ voxel size. Each mass was traced and a contour was created and evaluated for bone volume fraction (BV/TV), i.e. the ratio of bone volume over total volume. The 3D data sets were low-pass filtered using a Gaussian filter (*σ*=0.80, support=1) and segmented with a fixed threshold filter (160 mg HA/cm^3^). These tissues were then embedded in paraffin and sectioned at 6μm thick. The slides were stained with alcian blue or alizarin red and imaged on an Optiphot microscope (Nikon, Melville, NY) with a Spot Color Camera (Diagnostic Imaging,). To count cells in the ectopic ossification tissue, slides were deparaffinized, rehydrated, mounted with Vectashield Hard Set with DAPI, and imaged on an Eclipse E800 microscope (Nikon) with an Evolution QEi Monochrome camera (Media Cybernetics). Histomorphometric analyses were performed using Image Pro Plus 7.0 (Media Cybernetics) to measure a ratio of area positively stained by DAPI nuclei to total area.

### Statistical analysis

Statistical analysis between groups was performed using a one way ANOVA for normality and Student's *t*-test for continuous variables. A level of significance (*α*), or a *P*-value of less than 0.05, with a 95% confidence interval was determined. Representative data are presented as the mean±S.E.M. of 2-6 individual samples from 3 to 6 independent analyses. All data passed normality and equal variance tests and was analysed using a one-way ANOVA.

## Abbreviations

APAlk Phos: alkaline phosphatase
AP-1: activator protein 1
ASK1: apoptosis signal-regulating kinase 1
BAD: Bcl-2-associated death promoter
BAX: Bcl-2-associated X protein
BID: BH3 interacting-domain death agonist
BMP: bone morphogenetic protein
BSP: bone sialoprotein
BV/TV: bone volume to total volume ratio
Ca^2+^: calcium
CASP3: caspase 3
COL-X: collagen X
DAB: 3,3′-diaminobenzidine
DAPI: 4',6-diamidino-2-phenylindole
DAXX: death-associated protein 6
GOX: glucose oxidase
GP: growth plate
H2AX: H2A histone family, member X
H_2_O_2_: hydrogen peroxide
Het: heterozygous
HZ: hypertrophic zone
JNK: c-Jun N-terminal Kinase
KO: knockout
MAPK: mitogen-activated protein kinase
MEF: mouse embryonic fibroblast
MicroCT; *μ*CT: micro-computed tomography
MMP-13: matrix metallopeptidase 13
MTT: 3-(4,5-dimethylthiazol-2-yl)-2,5-diphenyltetrazolium bromide
NF*κ*B: nuclear factor kappa B
NQDI-1: selective small molecule inhibitor of ASK1
NS: not significant
Oc.S/BS: osteoclast surface on bone surface area
PARP: poly (ADP-ribose) polymerase
PHZ: prehypertrophic zone
PZ: proliferative zone
ROS: reactive oxygen species
RUNX2: runt-related transcription factor 2
RZ: resting zone
Staur: staurosporine
Tb.N.: trabecular number
TGF*β*: transforming growth factor beta
TRAP: tartrate-resistant acid phosphatase
VEGF: vascular endothelial growth factor
WT: wildtype
