# Supplementary figures and images for: Inhibition of apoptosis signal-regulating kinase 1 enhances endochondral bone formation by increasing chondrocyte survival
Source: Cell Death Dis. 2014 Nov 13;5(11):e1522–. doi: 10.1038/cddis.2014.480 (PMC4260738; doi:10.1038/cddis.2014.480)

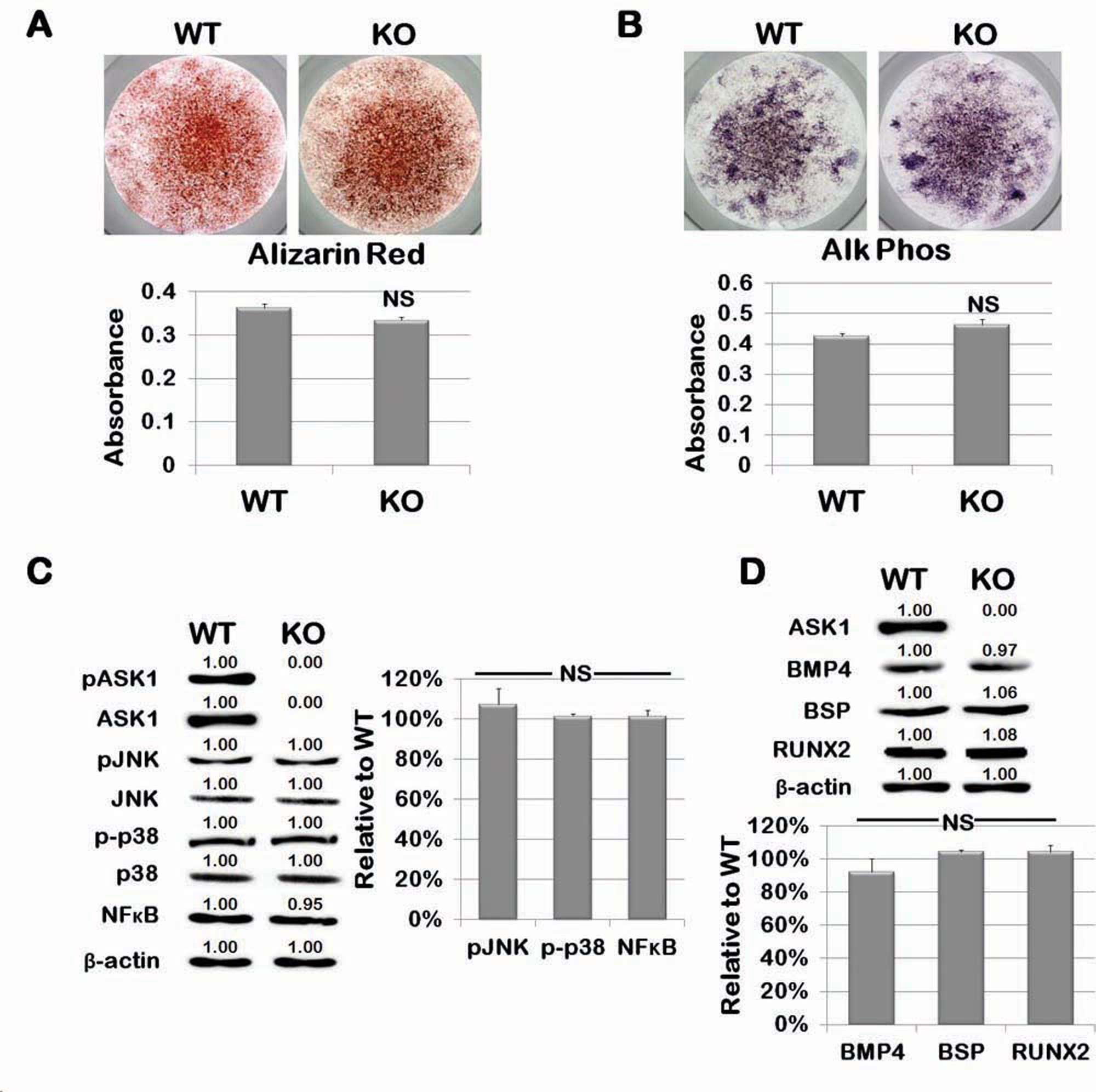

Supplement: Supplementary Figure 1 [file cddis2014480x1.tif]

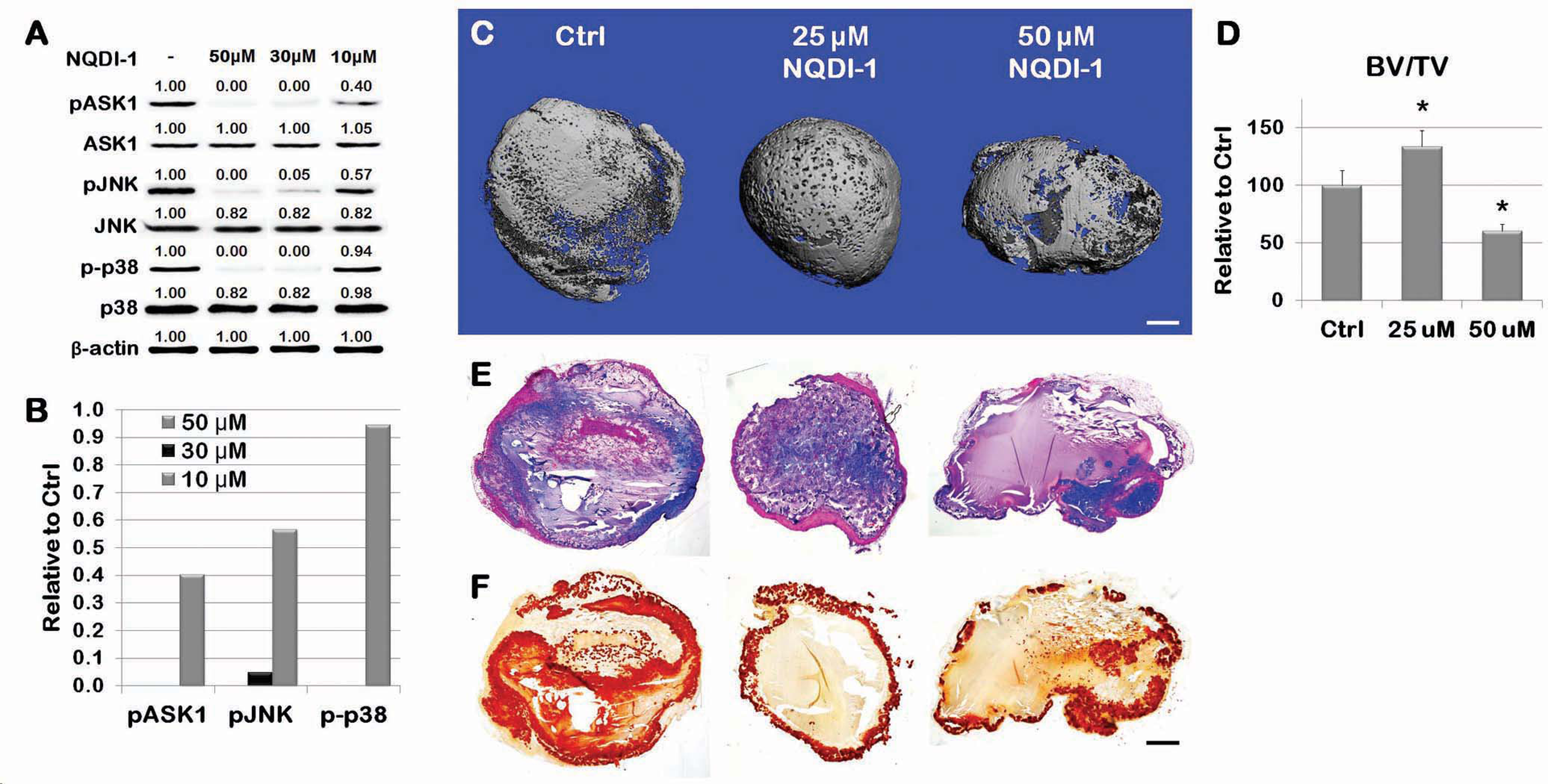

Supplement: Supplementary Figure 2 [file cddis2014480x2.tif]
