# Supplementary material for: Inhibition of apoptosis signal-regulating kinase 1 enhances endochondral bone formation by increasing chondrocyte survival
Source: Cell Death Dis. 2014 Nov 13;5(11):e1522–. doi: 10.1038/cddis.2014.480 (PMC4260738; doi:10.1038/cddis.2014.480)
Supplement: Supplementary Figure Legends [file cddis2014480x3.doc]

**Supplemental Figure 1. Similar osteogenic potential of WT and KO MEFs.** **(A)** Alizarin red staining of MEFs generated from WT and ASK KO mice, seeded as micromasses, and cultured in osteogenic media showed no difference in osteogenic differentiation. **(B)** Alkaline phosphatase staining of MEFs generated from WT and ASK KO mice, seeded as micromasses, and cultured in osteogenic media showed no difference in osteogenic differentiation. **(C)** Western blots showed no change in expression of ASK1 KO downstream activation of JNK, p38, or NFκB. **(D)** No change in expression of osteogenic markers BMP4, BSP, or RUNX2. (NS = not significant; micromass: n=12 wells for each genotype; Western: n=4 replicates for each genotype)

**Supplemental Figure 2. ASK1 inhibition by NQDI-1 in WT MEFs and ectopic ossification.** **(A)** A concentration curve to determine maximal ASK1 inhibitory concentration of NQDI-1 was performed with concentrations of 50µM, 30µM, and 10µM. **(B)** Western blot analysis of phosphorylated ASK1, JNK, or p38 showed sufficient ASK1 inhibition at 30µM NQDI-1, but insufficient at 10µM. **(C)** Matrigel /BMP subcutaneously injected in CD-1 mice alone or with 25µM or 50µM NQDI-1. **(D)** MicroCT analysis showed 25µM NQDI-1 enhanced bone deposition, while 50µM decreased bone formation, and serial sections stained with **(E)** alcian blue and H&E showed enhanced cartilage visualization and **(F)** alizarin red showed mineral deposition at all three concentrations. (n=5 for each treatment; * = p ≤ 0.05; ** = p ≤ 0.01; scale bars = 1mm)
